# Supplementary material for: Hypoxia promotes an inflammatory phenotype of fibroblasts in pancreatic cancer
Source: Oncogenesis. 2022 Sep 15;11(1):56. doi: 10.1038/s41389-022-00434-2 (PMC9478137; doi:10.1038/s41389-022-00434-2)
Supplement: Supplementary file 1 — Supplementary Information [file 41389_2022_434_MOESM1_ESM.pdf]

Fig. S1

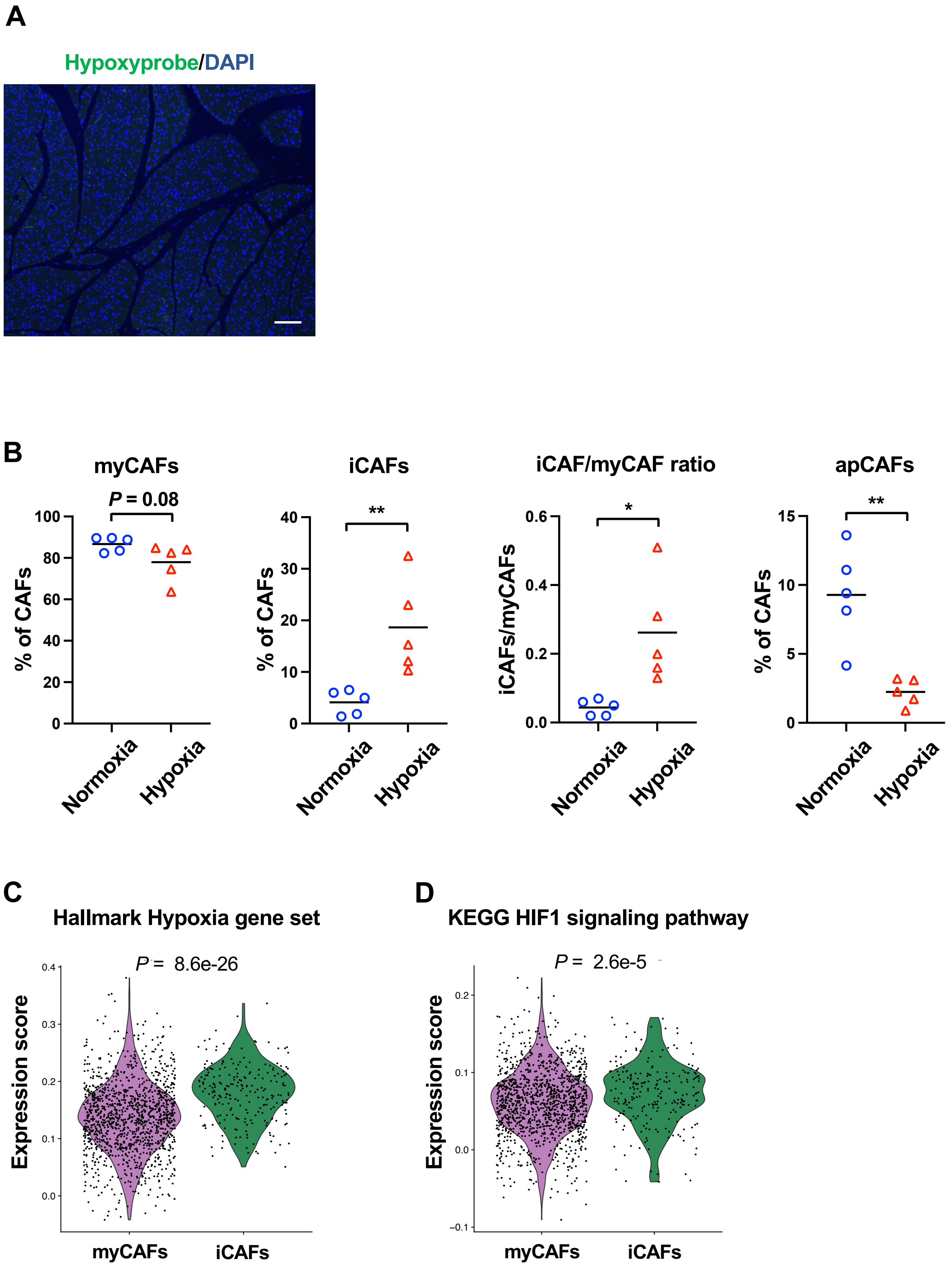
A
Hypoxyprobe/DAPI
B
myCAFs
P = 0.08
% of CAFs
100
80
60
40
20
0
Normoxia
Hypoxia
iCAFs
**
40
30
20
10
0
iCAF/myCAF ratio
*
iCAFs/myCAFs
0.6
0.4
0.2
0.0
apCAFs
15
10
5
C
Hallmark Hypoxia gene set
P = 8.6e-26
Expression score
0.4
0.3
0.2
0.1
0.0
myCAFs
iCAFs
D
KEGG HIF1 signaling pathway
P = 2.6e-5
0.2
0.1
0.0
−0.1

Fig. S1. Distinct CAF subset proportions in normoxic and hypoxic tumor regions.

(A and B) WT mice and mice bearing 4-week orthotopic PDAC received an intraperitoneal injection with 60 mg/kg of Hypoxyprobe and were sacrificed 1.5–2 hours later.

(A) Immunofluorescence staining for Hypoxyprobe (green) and DAPI (blue) in pancreatic tissues from WT mice. Scale bar, 100 μm.

(B) Percentage of myCAFs, percentage of iCAFs, iCAF/myCAF ratio, and percentage of apCAFs among normoxic and hypoxic CAFs from 4-week orthotopic PDAC of 4662 tumor cells, as analyzed by flow cytometry (n=5).

(C) Violin plot of Hallmark hypoxia gene set expression score in human myCAF and iCAF populations.

(D) Violin plot of KEGG HIF1 signaling pathway expression score in human myCAF and iCAF populations.

The symbols in (B) represent individual mice, and horizontal lines represent the means. P values were determined by student's *t* test (B) and Wilcoxon rank sum test with Bonferroni correction (C and D). $^*p < 0.05$; $^{**}p < 0.01$.

Fig. S2

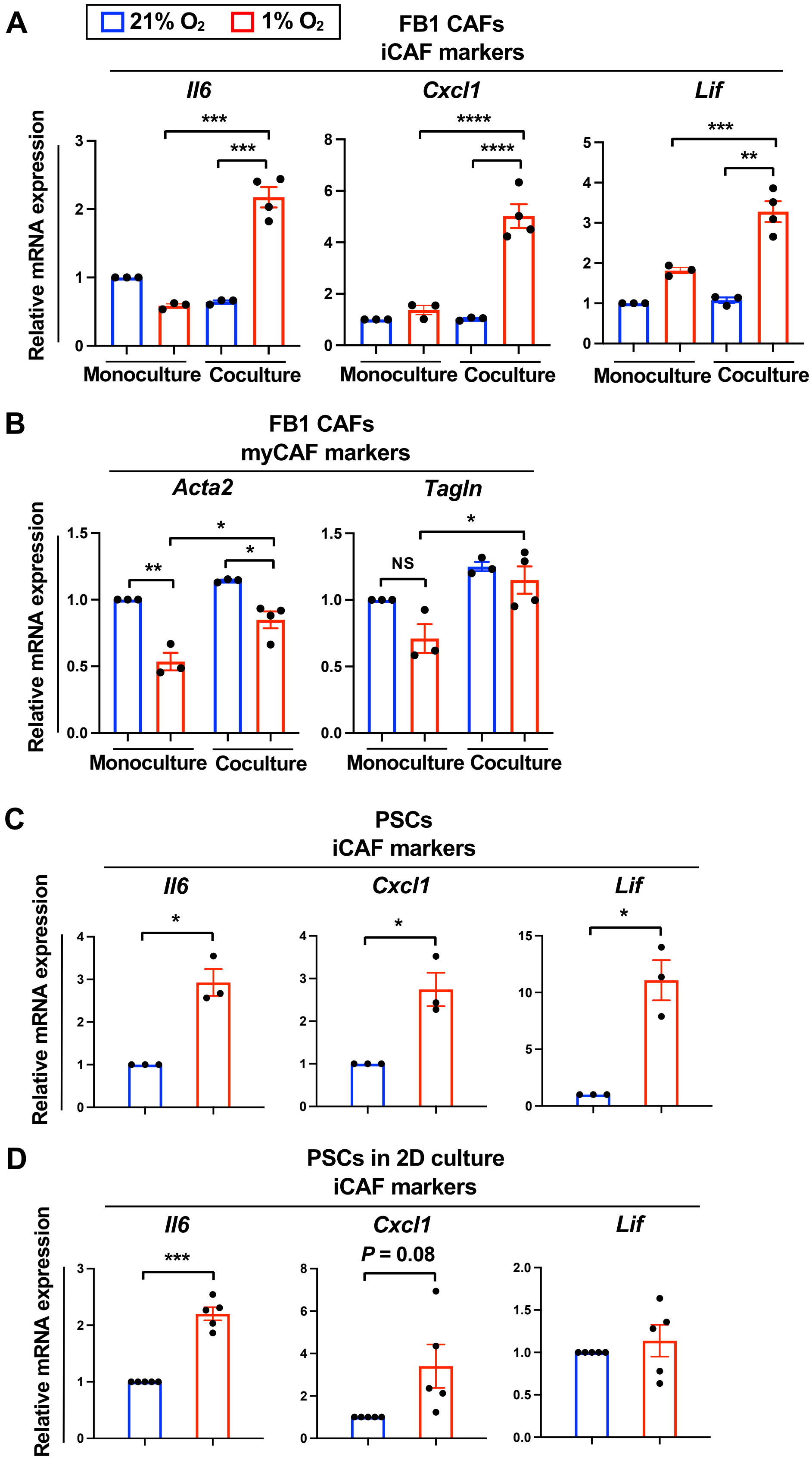

A
21% O_2
1% O_2
FB1 CAFs
iCAF markers
Il6
Cxcl1
Lif
Relative mRNA expression
***
***
****
****
***
**
Monoculture
Coculture
B
FB1 CAFs
myCAF markers
Acta2
Tagln
Relative mRNA expression
**
*
*
NS
*
Monoculture
Coculture
C
PSCs
iCAF markers
Il6
Cxcl1
Lif
Relative mRNA expression
*
*
*
D
PSCs in 2D culture
iCAF markers
Il6
Cxcl1
Lif
Relative mRNA expression
***
P = 0.08

E

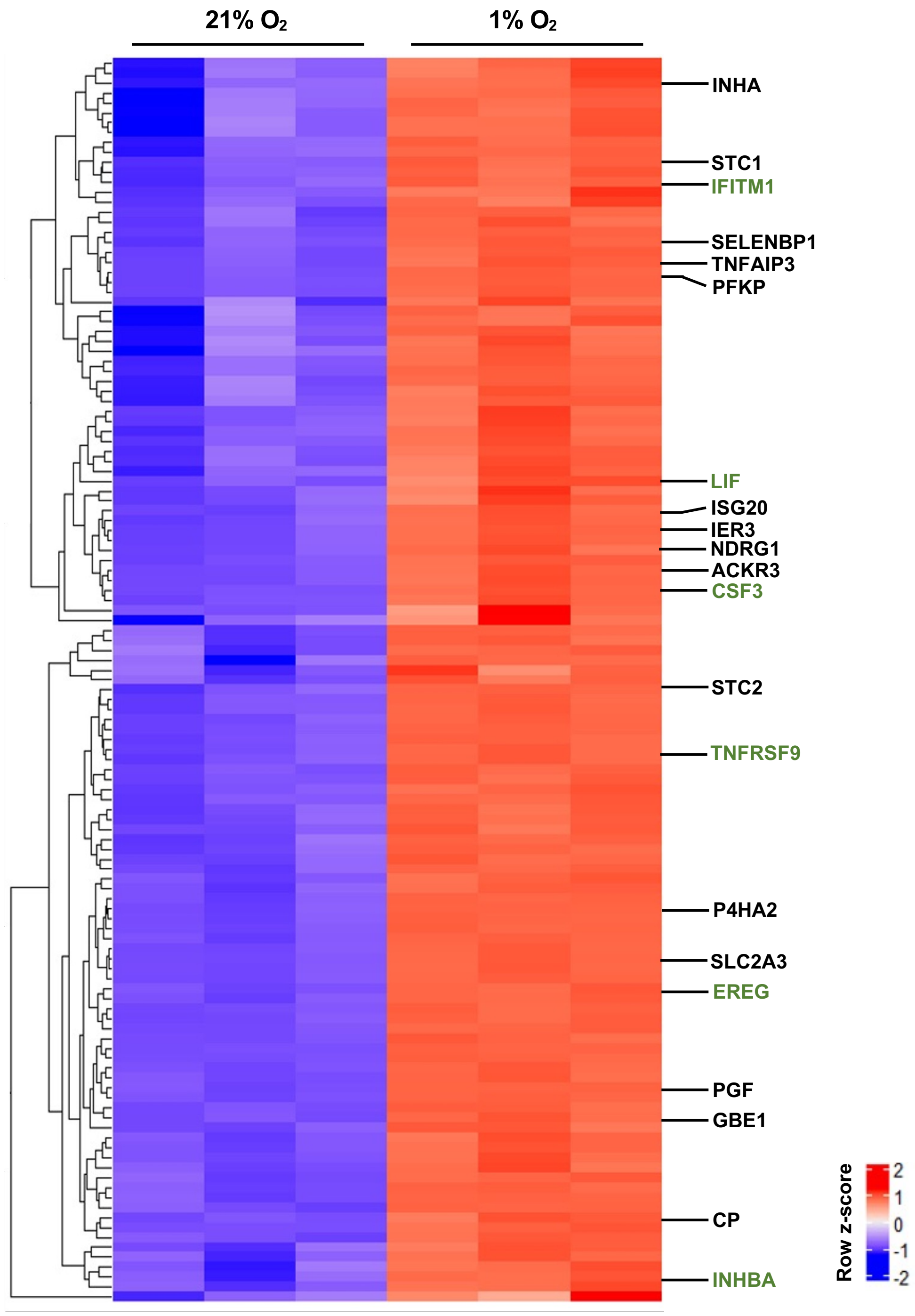
21% O_2
1% O_2
INHA
STC1
IFITM1
SELENBP1
TNFAIP3
PFKP
LIF
ISG20
IER3
NDRG1
ACKR3
CSF3
STC2
TNFRSF9
P4HA2
SLC2A3
EREG
PGF
GBE1
CP
INHBA
Row z-score
2
1
0
-1
-2

Fig. S2. Hypoxic induction of the iCAF phenotype.

(A and B) Quantitative RT-PCR analysis of iCAF (A) and myCAF markers (B) in CAFs (FB1) cultured alone or with mT3 tumor organoids under 21% O_2 or 1% O_2 for 72 hours (n=3-4). Expression levels were normalized by *18S rRNA*.

(C) Quantitative RT-PCR analysis of iCAF markers in PSCs cultured with 4662 tumor organoids under 21% O_2 or 1% O_2 for 48 hours (n=3). Expression levels were normalized by *18S rRNA*.

(D) Quantitative RT-PCR analysis of iCAF markers in PSCs cultured with mT3 tumor cells in 2D culture under 21% O_2 or 1% O_2 for 48 hours (n=5). Expression levels were normalized by *18S rRNA*.

(E) Heatmap showing 125 differentially expressed genes (color-scaled, row-wise z-scores) between the normoxic (21% O_2) and hypoxic (1% O_2) PSCs cocultured with PDAC organoids. The “hypoxia signature” and “inflammatory response” genes are labelled in black and green respectively.

Each data point in (C and D) represents individual primary PSC lines. Data in (A-D), mean±SEM. P values were determined by two-way ANOVA with Bonferroni post-test (A and B) and student’s *t* test (C and D). NS, not significant. $^*p < 0.05$; $^{**}p < 0.01$; $^{***}p < 0.001$; $^{****}p < 0.0001$.

Fig. S3

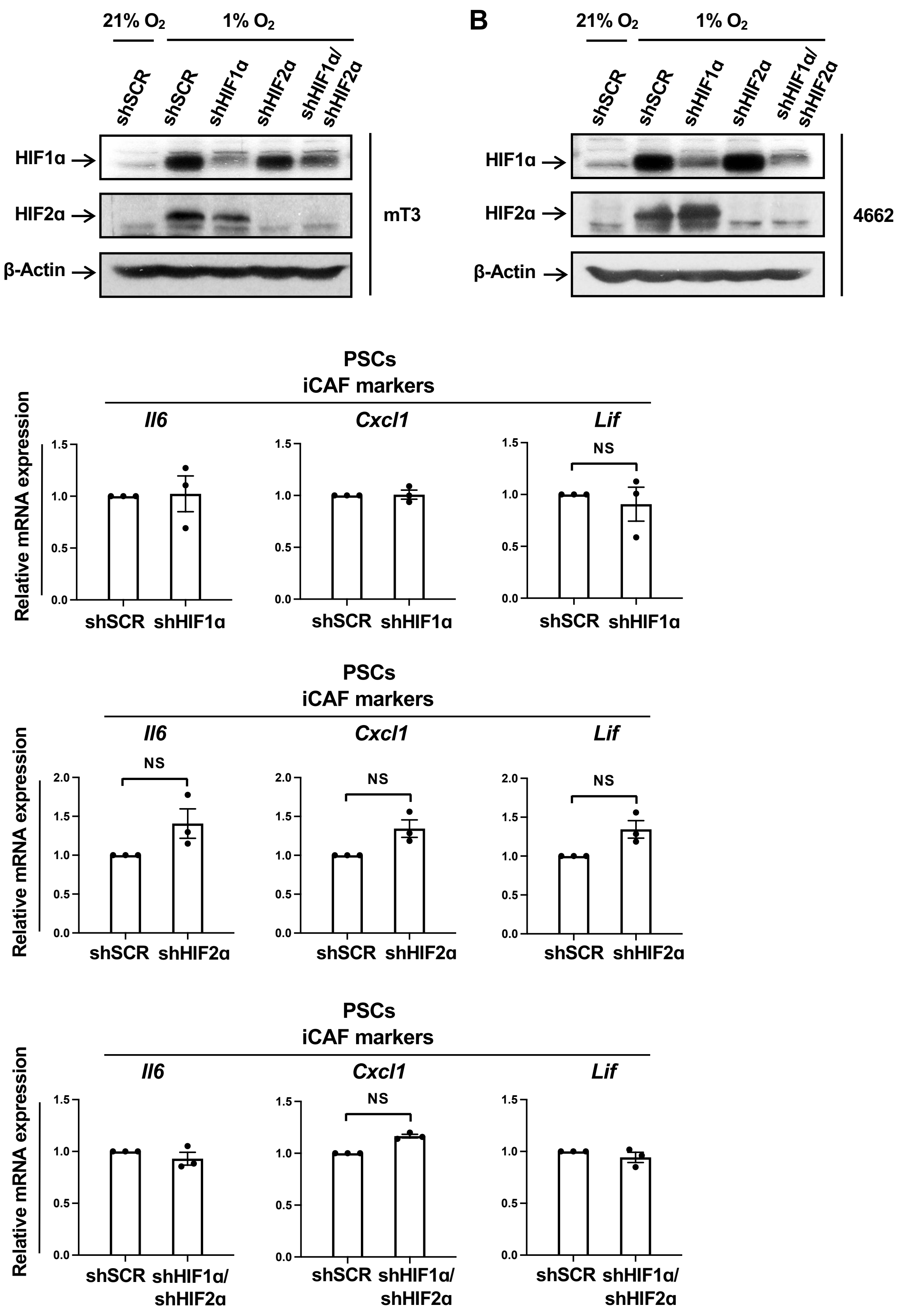
A
21% O2
1% O2
shSCR
shSCR
shHIF1α
shHIF2α
shHIF1α/
shHIF2α
HIF1α
HIF2α
β-Actin
mT3
B
21% O2
1% O2
shSCR
shSCR
shHIF1α
shHIF2α
shHIF1α/
shHIF2α
HIF1α
HIF2α
β-Actin
4662
C
PSCs
iCAF markers
Il6
Cxcl1
Lif
Relative mRNA expression
NS
shSCR
shHIF1α
D
PSCs
iCAF markers
Il6
Cxcl1
Lif
Relative mRNA expression
NS
shSCR
shHIF2α
E
PSCs
iCAF markers
Il6
Cxcl1
Lif
Relative mRNA expression
NS
shSCR
shHIF1α/
shHIF2α

Fig. S3. Knockdown of HIF1α and HIF2α in PDAC tumor cells.

(A and B) Western blot analysis for HIF1α and HIF2α in mT3 (A) and 4662 tumor cells (B) expressing shSCR control, shHIF1α, shHIF2α, or both shHIF1α and shHIF2α exposed to 1% O_2 for 16 hours. Equal loading was verified with anti-β-Actin.

(C-E) Quantitative RT-PCR analysis of iCAF markers in PSCs cultured with 4662 tumor organoids expressing shSCR control, shHIF1α (C), shHIF2α (D), or both shHIF1α and shHIF2α (E) under 1% O_2 for 48 hours (n=3). Expression levels were normalized by *18S rRNA*. Each data point in (C-E) represents individual primary PSC lines. Data in (C-E), mean±SEM. P values were determined by student's *t* test. NS, not significant.

Fig. S4

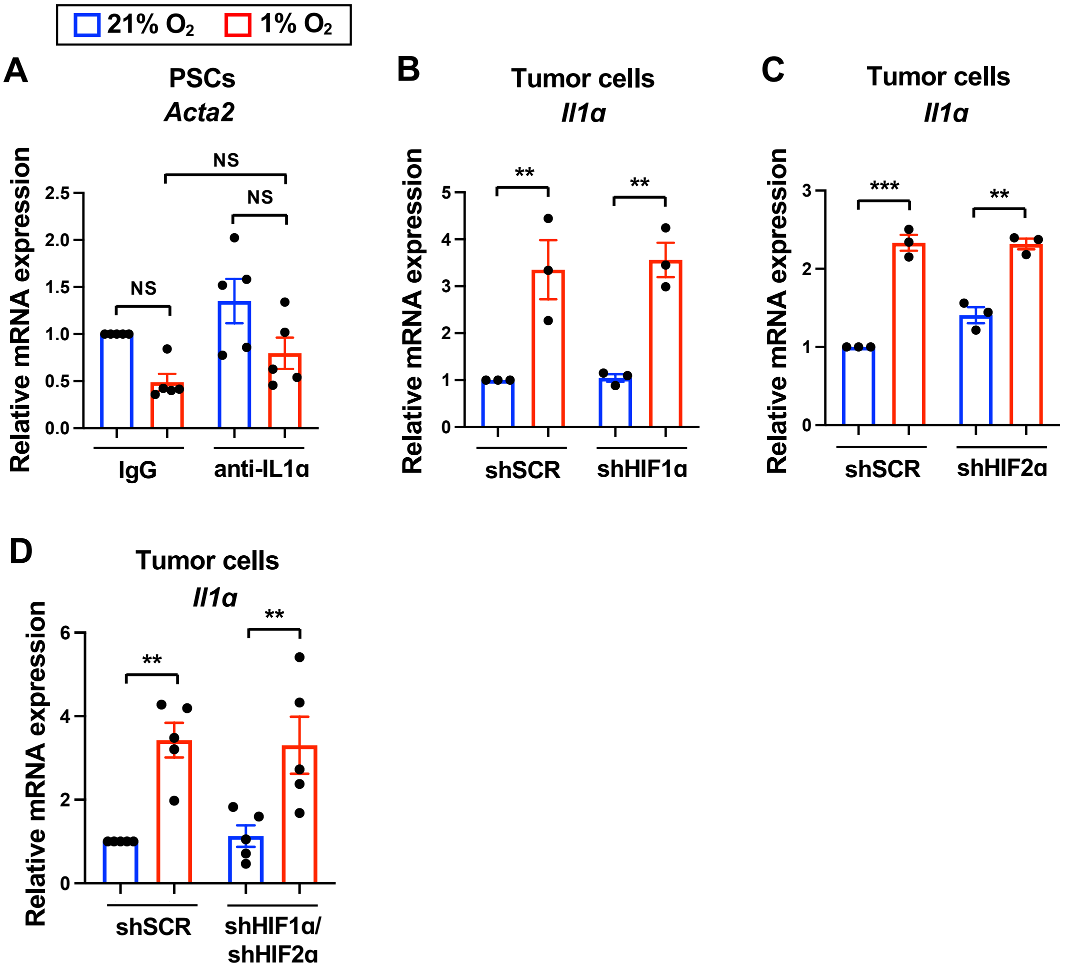

Fig. S4. IL1α does not significantly affect *Acta2* expression in PSCs and is regulated in a HIF-independent manner.

(A) Quantitative RT-PCR analysis of *Acta2* in PSCs cultured with mT3 tumor organoids in the presence of IL1α-neutralizing antibody or isotype control antibody under 21% O_2 or 1% O_2 for 72 hours (n=5).

(B-D) Quantitative RT-PCR analysis of *Il1a* in mT3 tumor organoids expressing shSCR control, shHIF1α (B), shHIF2α (C), or both shHIF1α and shHIF2α (D) cultured with PSCs under 21% O_2 or 1% O_2 for 48 hours (n=3 shHIF1α, n=3 shHIF2α, n=5 shHIF1α/shHIF2α).

Expression levels were normalized by *18S rRNA*. Each data point represents individual primary PSC lines. Results show mean±SEM. P values were determined by two-way ANOVA with Bonferroni post-test. NS, not significant. **p < 0.01; ***p < 0.001.

Table S1. Primer sequences for quantitative RT-PCR. Related to MATERIALS AND METHODS.

Gene	Forward Primer	Reverse Primer
18S rRNA	GTAACCCGTTGAACCCCATT	CCATCCAATCGGTAGTAGCG
Acta2	TGCTGACAGAGGCACCACTGAA	CAGTTGTACGTCCAGAGGCATAG
Cxcl1	TCCAGAGCTTGAAGGTGTTGCC	AACCAAGGGAGCTTCAGGGTCA
Il1α	ACGGCTGAGTTTCAGTGAGACC	CACTCTGGTAGGTGTAAGGTGC
Il6	TACCACTTCACAAGTCGGAGGC	CTGCAAGTGCATCATCGTTGTTC
Lif	ATTGTGCCCTTACTGCTG	TGTTAGGCGCACATAGCTTTT
Tagln	GCAGATGGAACAGGTGGCTCAA	CCCAAAGCCATTAGAGTCCTCTG
